# The homeless population during the COVID-19 syndemic: Inequities, practices of social resilience, and social reintegration strategies

**DOI:** 10.3389/fsoc.2022.959178

**Published:** 2022-09-30

**Authors:** Roberta Bova

**Affiliations:** Department of Human and Social Sciences, University of Bergamo, Bergamo, Italy

**Keywords:** homelessness, social misrecognition, social resilience, social reintegration, COVID-19 pandemic emergency measures

## Abstract

This paper analyses the amplification of social insecurity and the social misrecognition of the homeless during the COVID-19 syndemic. The research was carried out in the city of Bergamo (IT), which has been severely affected by the COVID-19 syndemic since the early months of 2020; the research was developed in two phases. The first one analyses the practices of social resilience activated during the COVID-19 syndemic by the socio-educational staff and the coordination figures who work in the support services. The second phase analyses the different social dynamics that can improve the wellbeing and social reintegration of the homeless from a long-duration perspective. During the first months of 2020, the public authorities failed to pay attention to homeless people who slept on the streets and who lived in communities or found support in night shelters. The support services had to activate immediate emergency response strategies and subsequently had to produce and purchase protective devices for operators, guests and those who remained on the street. Faced with this process of social misrecognition, the support services for homeless people reacted by activating practices of social resilience. These practices have investigated the dimensions of daily interactions and the symbolic and value configurations connected to them. However, directly conversing with the homeless, it emerges that to achieve full social reintegration and to prevent new forms of social misrecognition, in the event of future social or health crises, the relationship with a non-stigmatized social community is fundamental. Consequently, the primary objectives that the support network for homeless people should set for future projects should involve the local community through project participation activities and raise awareness of the phenomenon of poverty.

## Introduction

A wide field of interdisciplinary studies highlights how the homelessness phenomenon is multidimensional, including economic, social, psychological, health and political characteristics (Fitzpatrick, 2013). These dimensions come into play in the definition of the phenomenon, in the search for the causes that lead individuals and families to remain homeless, and also in the paths of reintegration (Fitzpatrick, 2013). The term homeless refers to a person whose characteristics fall within the European Typology on Homelessness and Housing Exclusion (ETHOS), or to a person who, in the absence of a structured and permanent residence, lives in a public space, in a night shelter or in an open place. Occasionally, the homeless are precarious guests of acquaintances and are forced to spend several hours of the day in an open space. The lack of a permanent residence often leads to the absence of a registered residence, and therefore, a lack of access to an important series of services guaranteed by the Italian state, including primary registration with the NHS (National Health Service).^1^

Regarding the analysis of the factors that determine entry into the homeless condition, there are two schools of thought. According to the sociological school (Anderson, 1923; Elliot and Krivo, 1991; Hopper and Baumohl, 1996; Wacquant, 2008), entry into the state of homelessness is determined by causes linked to the social context including problematic living conditions, poor distribution of national income, migratory displacement, a high percentage of single-parent families, of elderly people and finally, of young people who are unable to access the first job. The second school of thought, known as psychological (Bassuk et al., 1984; Snow and Bradford, 1994; Sosin and Grossman, 2003), emphasizes individual experience and therefore on a possible initial deficit or triggering event. While the hypothesis of an initial deficit (mental illness or drug addiction) has little evidence from research, the hypothesis of a triggering event is confirmed by numerous studies that show how the traumas caused by certain events can have repercussions on the management of life in general (Rizzo et al., 2022). In any case, the sociological and psychological approaches of the triggering event are not mutually exclusive; in fact, it is not necessary that any shocks or trauma precede entry into homelessness, and sometimes they follow it. On the other hand, the absence even for a short period of a stable home leads to precarious material conditions of existence and the disintegration of the social and emotional network (Tosi, 2005). This situation can concern those who have recently been released from prison or from therapeutic communities, for whom the prolongation of the period of isolation has led to the breaking of previous relational ties. In short, the absence of a home entails the lack of an environment dedicated to the development of stable emotional relationships, capable of carrying out the so-called buffering effect (Cohen and Willis, 1985). This buffering effect can absorb the stress caused by traumatic events or situations experienced as very tiring.

The contemporary debate regarding the strategies to be adopted to counter entry into homelessness and promote social reintegration sees public institutions and organizations that work with homeless people, as well as university research, among the main players. Some problematic issues emerge from this debate. The first issue is linked to the presence of a large number of homeless people who systematically avoid coming into contact with the network of services. Often, these are new users who fear being severely stigmatized, despite finding themselves in a situation perceived as “passing through” (Christian and Abrams, 2003). Another situation is that of homeless people who for several years have used the network of services and matured into a condition of chronicity and dependence on social assistance, failing to undertake social reintegration paths (Tosi, 2005). Both of the situations noted call into question the relationship between the homeless person and the social networks of reference.

It is still very difficult to quantify the homeless phenomenon, both locally and nationally. In Italy, a national census is underway, which also includes the homeless, among the “special populations”. To count the homeless, the National Institute of Statistics (ISTAT) has developed a method of data collection based on the registers of the reception structures, the support services and the municipal registry. From the latest ISTAT survey^2^ on the homeless population, dating back to 2014, it is estimated that in Italy there are almost 51,000 (equal to 2.43 per thousand of the total population) people without a stable home. The survey also noted an increase in the share of people who remain homeless for more than 2 years (41.1%) and 4 years (21.4%). These are mostly men (85.7%), foreigners (58.2%), under the age of 54 years (75.8%), or with low-educational qualifications (only one-third reached at least a secondary school diploma). In recent years, there has been a decrease in the number of foreigners aged under 34 years residing in Italy; therefore, the average age of homeless people has also increased slightly (from 42 to 44 years). A significant percentage of the homeless population is concentrated in the northern regions (56%), where the province of Bergamo is located. From the latest systematic survey regarding the province of Bergamo in 2017,^3^ it appears that 834 people were received at first reception facilities,^4^ of which 74% were foreign nationals and 94% were male. In this area, reception and support services and structures have been organized for many years. For example, in the field of prevention and addiction, there are the street units of the Addiction Service and two help desks, one by the municipality and the other managed by a charity association. Additionally, other first aid services are managed by some charitable associations, for instance, a nocturnal shelter. In this service, there is a significant turnover since most shelter recipients last <3 months; only in a residual percentage of cases, about 20% of homeless people move to other types of social accommodation. Examples of other social accommodations are therapeutic communities and social housing. In recent years, there has been a significant increase in the homeless population in Italy and Northern Italy in particular. The value has increased compared to 3 years earlier when it was 2.31 per thousand (equal to 47,000,648 people). Furthermore, a growing part of the homeless population is represented by Italian citizens.

The constant increase in a segment of the population of conditions of poverty and serious social marginality may be associated with an increase in the cost of basic necessities and the contraction of the labor market (Benassi et al., 2020). These are some of the effects of the economic and financial crisis of 2007–2008, and also the American real estate bubble of the early 2000s, during which stock prices fell sharply, as did global GDP. State intervention, to avoid a systematic crisis, led to an increase in public debt and the start of a spiral of recession. In the following years, financial institutions tightened their credit conditions, and this led to a general decline in business activities, as well as in the consumption of many households. The crisis led to an exacerbation of inequalities. The income of the super-rich increased and, at the same time, the number of families in conditions of social vulnerability and poverty grew (Tooze, 2018).

However, we can also mention some more structural dynamics, such as the precarious situation of the main systems of social integration and resource distribution (Bifulco and Vitale, 2006; Muehlebach, 2012). In particular, in the countries of southern Europe, such as Italy, there is a specific welfare system (Ferrera, 1996; Allen, 2006) that was created after the Second World War when a high percentage of the economy was still rural, slowing down the urbanization process. The family retains a decisive role in the public sphere, and the state supports conservative and liberal political action, with an anti-communist attitude (Ferrera, 1996). In this context, social housing policies straddle different contexts: the private market and public action. The private market plays a decisive role, given the high proportion of owned properties in Italy (equal to 76% of real estate assets) (Allen, 2006). The dominant role still played by Italian families in supporting the purchase of homes for young people and couples seems to compensate for the poor development of the financial sector, as well as the precariousness of the labor market. The supply of public rental housing is also very low compared to the demand, and it is also excessively targeted and therefore cannot have a significant effect on social hardship (Bifulco and Vitale, 2006). Added to this is the clientelism characterizing the public bureaucratic apparatus (Allen et al., 2004). All these factors produce an enlargement of groups of the population in conditions of poverty or social vulnerability (Benassi et al., 2020), for whom public policies have so far not been able to develop effective responses (Ranci and Pavolini, 2008; Kourachanis, 2021).

During the twentieth century, European states developed social protection systems based on a dynamic labor market, standardized life models and family systems, and, above all, economic benefits and social safety nets that acted as social safety nets (Ranci and Pavolini, 2008). The combination of these factors could guarantee economic wellbeing and favor the spread of a sense of security and self-confidence, as well as the ability to invest in the future (Beck, 1986). With the emergence of the neoliberal paradigm during the 1980s, initially, in Britain and the United States (led by Margaret Thatcher and Ronald Reagan, respectively) and then in most other states in the western hemisphere, the traditional welfare system was progressively eroded (Harvey, 2005). The logic of market efficiency requires maximum freedom of action, to the detriment of political and social forms of regulation (deregulation). The promise is that deregulation should gurantee profit and wellbeing for nations and individuals. Over the decades, however, it has become clear that neoliberal politics have produced increases in inequality, exploitation and pollution (Stiglitz, 2019). Moreover, the neoliberal approach seems to transmit an idea of individualistic social risk. As society has disappeared—to quote Margaret Thatcher—attention has become focused only on personal responsibilities in the management of social risk (Hamilton, 2014). This structural change to the capitalistic economy has caused a reorganization of the welfare model in European countries (Kourachanis, 2020). Actually, after the creation of the European Stability Mechanism in 2011, the dominant philosophy of social policy has become cost containment to guarantee the sustainability of the welfare services (Moreno, 2016). Examples of such policy shifts are the extension of targeting in policy provisions, the de-universalization of benefits and services, and the introduction of labor activation (Lister, 2011). This model of social policy promotes the responsibilization of each individual who benefits from social citizenship, but it could increase the social exclusion of the poorest and least powerful in society (Lister, 2011). Living conditions characterized by prolonged precariousness (Consoli, 2019), anxiety and uncertainty seem to be gradually becoming widespread (Magatti, 2012). Today's threats seem to be derived from the same social fabric (Giddens, 1991) and from a technocratic approach to social planning (Lusardi and Tomelleri, 2021).

This “risk society” (Beck, 1986) seems to have direct repercussions on the social status of the subject, and on the right to access protection connected to the basic needs of the person, which is now administered according to market mechanisms (Dubois, 2003; Lenoble, 2014), to the detriment of the traditional safeguards guaranteed by state law.

It is the contemporary dynamics of work that produce a “supernumerary” segment of the population (Castel, 1995), people who are endowed with inadequate professional skills and are constantly available in the labor market for temporary and poorly paid jobs (Castel, 2007); these constitute the so-called working poor.^5^ For the homeless population, the gradual loss of density of family networks and primary sociality, as well as poor public recognition, are added to their problematic insertion into the labor market (Acosta and Toro, 2000; Wong and Piliavin, 2001; Barker, 2012).

The homeless person often experiences partial participation in the social context, both at the institutional level (in various situations, public and health institutions pay little attention to the protection of the homeless person), and in the context of civil society (Bourgois, 2003; Mitchell, 2003; McNay, 2008). When feeling himself or herself to be a member of a social context, an individual places himself in dialogue with the expectations that the other members have of his behavior and the social roles he interprets (Honneth, 1996). The challenge that a person faces in the course of his life is therefore that of distinguishing himself from others while deriving this distinction from his own recognition (Sennett, 2004). Social recognition seems to be a determining factor in the social construction of individual identity, and it contains within itself different degrees of social participation, such as interaction and participation in the institutional and political context. Honneth (1996, 2004) identifies three phases of the conflict–recognition–conciliation process. The first occurs between children and parents, but also between friends or partners, and has a predominantly emotional character. The second phase concerns the recognition of individual rights that allow people to participate freely in the labor market. Finally, the third phase is constituted by living in society and participating autonomously in social solidarity. In the three phases, the subject passes from needing recognition, in the infantile phase, through a second phase of suspension in which he assumes the characteristics of an abstract bearer of rights, to the third phase in which he identifies his own uniqueness and, on the basis of this, participates in society. When this process does not take place and the subject fails to participate in social solidarity, there has been a misrecognition and, consequently, he is unable to rework his identity fully and achieve complete autonomy (Honneth, 1996, 2004; McNay, 2008).

The process of social misrecognition can take different forms: physical violence, legal exclusion and offenses to human dignity. These are experiences that affect the person as a violation of the intuitive principles of justice, and they make him aware of the injustice suffered. Homeless people daily experience forms of social misrecognition carried out by both bureaucratic apparatuses and civil society (Bifulco and Vitale, 2006; Wacquant, 2009). An example is the lack of recognition of the right to a registered residence and, therefore, the partial participation in territorial welfare services; another is the daily forms of stigmatization that prevent participation in the labor and housing markets (Mitchell, 2003; Tosi, 2007; Nolan, 2009).

The health emergency caused by COVID-19 has further highlighted the social exclusion affecting the homeless population, who, more than others, have been exposed to the risk of contagion (Barbieri, 2020). Even at the time of this health emergency, public and health institutions have paid little attention to the protection of the homeless population (Barbieri, 2020; Wang et al., 2021). As Horton (2020a,b) rightly observes, public and health authorities in the first months of the pandemic paid little attention to the protection of the social and health personnel involved in giving assistance to sick people, and concentrated their actions exclusively on the medical containment of the pandemic. However, after a more careful analysis, it soon became clear that the COVID-19 crisis should be interpreted as a syndemic (Horton, 2020b), in which biological factors interacted with social ones in determining the degree of risk to which people were exposed. The social groups most exposed to the risks derived from the COVID-19 crisis were the elderly, African-Americans, ethnic minorities, and the poor and precarious workers. To protect these people, a biomedical approach would not have been enough: the use of social action tools was also needed (Silva and Smith, 2020).

The difficulties that the European countries have shown in the initial management of the COVID-19 crisis are tangible clues to the structural weakness of the pre-existing dominant philosophy of social policy. Cost-containment, responsibilization, social misrecognition and individualistic social risk have been some of the determinants that have systematically lessened the containment of the syndemic.

However, the same health emergency has made it possible to bring out resources of social resilience within the network of services, which until now had not been identified and exploited, to combat serious adult marginalization. By social resilience, I mean the forms of adaptation of organizational resources and the transformation of symbolic configurations, professional practices and daily social interactions (Adger, 2000).

These resources were able to restore a situation of wellbeing and security. In any case, as emerges from a second examination (this time, carried out in direct dialogue with homeless people engaged in a process of social reintegration) to escape from social exclusion and overcome an emergency intervention approach, the challenge seems to be, rather, to create permanent conditions of the social recognition of diversity and to put in place policies aimed at developing a more welcoming and tolerant community context capable of encouraging and weaving new social bonds.

## Materials and methodology

The fieldwork research was based on a qualitative methodology and was developed in two phases. The first phase was conducted in the months of May–September 2020. I carried out 18 discursive interviews with the socio-educational staff and the coordination figures who work in the support services and reception facilities for the homeless. Theoretical sampling was opted for that reflected both the heterogeneity of professional profiles and the organizational structure of the services. A total of 18 interviews were carried out with 12 women and six men, of which: four were service coordinators, four were street workers of the Services for Pathological Addictions, eight were social educators working in therapeutic communities or night shelters and two were municipal social workers. The interviews analyzed the practices of social resilience (Adger, 2000; Keck and Sakdapolrak, 2013) activated during the COVID-19 syndemic and illustrated the changes that occurred in the organization of work, routines and habitual relational modalities during the syndemic crisis. The interviews were carried out in person, at the service offices and lasted around 90 min.

The second phase of the research was conducted in the months spanning January–September 2021. In this phase, I carried out 12 interviews with homeless people who were participating in social reintegration programs. Additionally, theoretical sampling was applied that reflected the heterogeneity of the homeless population residing in the province of Bergamo in terms of age, gender and nationality. The sample was composed of six men and six women; eight of them were immigrants and the average age was 36 years. This second group of interviews investigated the different social dynamics that can improve the wellbeing and social reintegration of the homeless. I focused my attention on the processes of stigmatization, disparities in the labor market, access to housing and the impact of social and family networks. The interviews were carried out in person, at the communities, in the dormitories, or affiliated accommodation, and lasted around 90 min.

The empirical material was transcribed in digital format and analyzed according to a procedure of theoretical coding and constant comparison of the analytical categories (Glaser and Strauss, 1967) to identify and isolate the main practices of social resilience and the processes in favor of social reintegration. In this report, a confidentiality protocol was applied which provided for the alteration of personal data and sensitive information to ensure the anonymity of the subjects involved.

## Results

### The health emergency and social exclusion of the homeless

The spread of the COVID-19 syndemic, which affected the Bergamo province in a particularly violent way since the early months of 2020, caused outbreaks of contagion in some residential services, as well as an increase in hospitalizations and deaths. These phenomena triggered the attention of the coordinators of the reception facilities for the homeless, aware of the health vulnerability of homeless people (Barbieri, 2020). Moreover, as a coordinator declared, “at that moment the institutions responsible for protection failed”. Many street workers reported having worked for the first month of the syndemic without the necessary safeguards:

In the first few months we all got sick and so we didn't see our colleagues for 15 days. Upon returning, we did not do any tests, serological or swabs; the operators of the Services for Pathological Addictions who do our same job have closed and swabs, for us nothing. And then you say “Okay we have to be there, but we have to get sick and make sick no thanks. Our users are homeless and it's not like they have to die”.

The lack of protection increased the tension toward the Local Health Care Area (ATS in Italian); as an educator reported, the impression was that “as the problems arose, they (ATS) were a little unloaded on us”, without the due assumption of responsibility toward the therapeutic communities; indeed: “ATS really threatened us in writing that if we did not guarantee service standards and in the event of a sudden inspection, they would shut us down”. As a coordinator told us, she felt “more precept than supported by ATS”.

Often, before the official indications from the government, the homeless services produced new regulations themselves: the canteens were replaced by the distribution of single-portion kits; the therapeutic communities suspended the entry of outsiders and limited the exits of their guests; the dormitories extended their opening to 24 h, reorganized the spaces to decrease the density of guests; the Services for Pathological Addictions reduced their opening hours; home educators replaced their visits by video calls; finally, a new structure was opened for the most unstable people, who used to sleep on the street.

The reorganization just described ensured new stability for the homeless services' network, in addition to this, it transmitted a sense of safety to the subjects involved. This feeling however could not be completely contrasted with the perception of having been “abandoned”. A coordinator described his community as “a medieval castle perched in itself”. Many coordinator interviewees underlined the lack of collaboration on the part of the institutional members of the homeless services' network (municipality, Local Health Care Area, politicians). The institutional bodies decided to interrupt the in-person interviews and reorganized the communication only by telephone and e-mail, just with the coordinators. The public services also decided to interrupt all paths of social reintegration, such as the start of an internship, the achievement of a disability allowance, and the entry into a therapeutic community. The social services of the municipality decided to concentrate their actions on new types of users, such as lonely elderly people and not self-sufficient people, and deliberately delegated the management of the homeless to the third sector and charity services. As a municipal social worker admitted: “those who were not in accommodation facilities were less protected”.

Local Health Care Area services also decided to close access channels and this resulted in serious psychological consequences for users, as a social worker of the Services for Pathological Addictions said:

The closure of various services has reduced the possibility of reaching people. It was difficult to manage the patient's expectation and having to tell him that everything was blocked; there was a kind of frustration to deal with: for someone who was ready to enter a council house, community or start a job, seeing these things suspended indefinitely made them angry: they didn't know if those things were going to be confirmed or if they jumped.

The health problem took precedence over the other areas of action: educational, social, economic, legal and relational (Folgheraiter, 2020; Horton, 2020b), as an educator said: “everything stopped and we had to give up the most important thing for our guests: the possibility to establish a relationship”. Even the street workers of the third sector services questioned themselves about their own protection, but after a week they realized that the famous slogan “I'm staying at home” for the homeless was out of the question. As social workers interviewed said “the people on the street are there, the need is there”; consequently, they decided to reopen the service, because “the frailties of street people have not stopped, indeed they have increased”.

As the coordinator of a street worker service assumed:

There were days when everything was closed, the person came to us and began to complain: “The municipality is closed, I had to meet the social worker, I want to go to the community and I can't I do more”. It was a moment when we felt bewilderment and we operators found ourselves doing, in addition to our work, also as consultants, doctors, nurses; users made every request to us, but simply because we were there and we were their only support.

Especially for that residual part of the most unstable users, who have active addictions or psychiatric problems, and could not respect the confinement inside the dormitories, the presence of street services' workers had fundamental importance to avoid the realization of “a sort of natural selection” for those who seemed to have been considered “waste lives” during the COVID-19 emergency (Bauman, 2003), deprived of fundamental health and legal protections.

### Practices of emergency resilience and the establishment of a social space of proximity

Faced with the health emergency, coordinators and educators were able to quickly reorganize services. First of all, given the lack of collaboration on the part of institutional bodies, the third sector network proved fundamental, from a practical point of view, to “maintain a common line”, and also to perceive a sense of “closeness, on the human side”, as a coordinator explained.

In a few days, shifts and roles were completely revolutionized, putting into practice the ability cited by an educator to “change clothes” and “put oneself at the service of the users”; this did not prevent coordinators and educators from maintaining a high degree of freedom and “to modulate their own line of action independently”, as a home educator remembered. The hourly flexibility also affected those who were normally “absorbed by bureaucracy” by carrying out coordination functions:

During the first weeks of the emergency we had to meet the needs of the facility to cover the absences of sick colleagues, not only educators, but also those in charge of the kitchen, cleaning, garden and therefore, we made ourselves available, obviously at personal discretion, in order to guarantee basic services.

The prolonged sharing of time and space between colleagues was an incentive for the development of a relationship dimension and the sharing of responsibilities. As an educator explained, every day they put into practice “the competence to trust and rely on others, think about the other as someone useful and indispensable for managing the situation”, adopting a systemic and group-focused gaze.

The elements that seem to have created the bridging function (Putnam, 2006) between staff members and users and prevented any conflict, seem to have been above all the personalization of the intervention, or the willingness to “have a not too technical gaze, but one attentive to detail”, especially in community work:

The core of our relationship work was this: to find a balance between the quality of the educational relationship and the distance imposed. There are things I do for you, user, but not necessarily with you. The metaphor is to remove the arms and legs, but still try to feel the heart: there was little to do, the hands weren't much use, but you still had to be able to make your presence felt. It was important to say every day: “Good morning” and to maintain small gestures of care.

The operator–user relationship did not conform only as a personalized help intervention (Sennett, 2004), responding to small needs and the institution of familiarity and trust, expressed by the metaphor of “taking hands” and “discovering pieces of history in more” of their experiences, as an educator told us, but also as a process of activating users' capabilities through direct participation in the administration of the structure (Sen, 1999; Nussbaum, 2011). As a community educator remembered:

The collaboration with the guests has increased a lot, also as regards the care of the common spaces, they had this extra attention, as if they felt at home; for example, there were guests who woke up in the morning and took care of the flower beds, tidying up the spaces. We also started listening to users a lot more and this has allowed us operators, to better understand the resources and the facets of each user.

Online Italian courses, painting, ceramics and gardening workshops were organized in small groups, also, as simple games of cards or preparing meals, such as unstructured moments of sharing and comparison; as a coordinator explained: “an exceptional normality was created: what was done was done together and decided together”. Finally, it was essential to empower the guests by conveying the idea of being a key element for the success of coexistence and overcoming the emergency. Community regulations had declined with a high rate of flexibility:

The rules have become less strict: those who arrived late could not be dismissed as before, but we had to create a relationship to make them understand how important it was to respect the rules for the wellbeing of all. With the guest it was said: “Now that we have explained to you, if you want to stay with us, if you want to go on your own it is your personal responsibility”.

Ultimately, the third sector network was able to activate new procedures, but above all to increase resources already present in everyday life; “a mix of change and permanence”, experienced in years of informal territorial collaboration between the various entities, only recently coordinated by the municipality. This networking process, although it presented some problems, seems to be at the basis of the constitution of a social space of proximity (Keck and Sakdapolrak, 2013; Folgheraiter, 2020). The absence of continuous and operational collaboration on the part of public bodies was particularly felt and as a social worker explained: “the very fact that we have noticed this lack, must lead us to make the things better from here on”. In this regard, among the coordinators interviewed, some admit that over the years they have been less attentive to the political side since they concentrated almost exclusively on the practical one:

We are very good at being with the last ones, but we are less good at being on the net and therefore, a discussion has started on how to create more systems. We still have to learn and the COVID-19 crisis has given us the opportunity to think about how to keep the political side, the head of the organization, inside the more technical tables; often the arm comes before the mind and sometimes the mind doesn't follow you.

Other respondents also identified a possibility in emergency management: first of all, to enhance one's work and: “give dorm work the right valor, while previously it was underestimated”, as an educator said. In addition to this, they expressed the expectation that some of the emergency resilience practices remain as learning available to the network:

We have certainly understood that “we can do”, in short, we are able to open up to slightly higher dynamics. Now it is useless to chase after all the changes we have experienced in recent months, but we are aware of saying: we have already put it into practice so the possibility is there and there is not always an emergency to put into practice the things.

In general, there seems to be a “greater circularity”, as stated by a coordinator, that is, a stronger sense of belonging and a greater sharing of activities and responsibilities. An awareness of the importance of the network also developed on the part of most institutional actors, “to be maintained and activated in case of need”, as explained by the social worker of the Services for Pathological Addictions.

Despite the abandonment by the institutions and the interruption of the reintegration paths, most of the operators were surprised by the resilience of the homeless and some interpreted this attitude as the result of years of work spent in building dialogue and a constant relationship between operator and guest and the outcome of the educational work put in place (Adger, 2000; Keck and Sakdapolrak, 2013). In addition to this, the responsibility of the guests was fundamental: no one was forced to stay in the structure, and in general, night shelters and communities tried to maintain flexible rules to avoid the creation of a “natural selection”. As a coordinator explained: the most unstable guests with active addictions or with behavioral problems would have preferred to give up the possibility of having a safe place to sleep and spend the day, rather than adhering completely to the new rules. In fact, few people have stayed in the dorms.

### Toward processes of social inclusion

As we have seen in the previous paragraphs, the health emergency initially reaffirmed the social exclusion and disregard of the homeless (Bauman, 2003; Wacquant, 2009). However, the ability to react and the social resilience practices expressed by the third sector network made it possible to restore wellbeing in the reception areas by providing a new awareness of the importance of social work, understood as a set of methods, attentions and practices (Keck and Sakdapolrak, 2013; Folgheraiter, 2020). Despite the important result achieved, direct dialogue with homeless people shows how, with the persistence of the health emergency, the difficulties of social reintegration and the risk of suffering new forms of social misrecognition persist. Although public and health, institutions have gradually become more attentive to the needs of homeless people, especially thanks to the solicitation of third sector subjects and the work of university research, the hardest obstacle to overcome seems to be represented by the difficulty in activating processes of social reintegration within the communities to which they belong (Acosta and Toro, 2000; Mitchell, 2003).

Often, from the stories of homeless people, who for years have been engaged in paths of overcoming grave marginality, the difficulty emerges of finding or rediscovering family networks, friends and neighborhoods available to welcome people in their frailty and to reshape previous relationships on the basis of the personal resources that each subject is able to put in place.

The economic crisis caused by COVID-19 led to the disappearance of many jobs in the informal economy in which homeless people often found employment; then it becomes really difficult for homeless people, who may have already exceeded 40 years of age, and did not have higher education qualifications and had years of unemployment behind them, to plan something solid and lasting (Benassi et al., 2020). Many users interviewed were aware of this, for example, a homeless woman from Marocco expressed this concern:

I don't know what will happen in my future, I've thought about it a lot, but I don't know. What I see is a bestial effort because with the job situation that exists now you have to work like an animal to get by, my son and I we will have to commit, I will have to do cleaning, cleaning and cleaning to make me exploit! And I still see charity, for quite a few years, even if by now we are becoming many who need it, and charity too is no longer behind it. I hope I can reuse the experience I'm having here, my internship at the greenhouses, maybe I find a job in agriculture, and not cleaning the offices.

This woman was aware of the current situation on the labor market, which had worsened with the COVID-19 crisis. She was also aware of the fact that it would be difficult, for the first few years at least, to be completely independent from the support services and that it would therefore take several months, if not years, to rebuild the economic security that allowed one to remain completely independent (Fraser and Gordon, 1994). In addition to this, the woman interviewed was also worried about her emotional state, in fact, despite maintaining a good relationship with her son, she expressed the fear of being alone and therefore, of being demoralized. When asked which mood she will feel, when she will be outside of the community, she replies:

So empty, because sometimes I think about this too, here after a while it becomes like a family: we have breakfast together, we all sleep together; the giggle before falling asleep, chatting, comforting, are things that do not give you a sense of loneliness and so you do not go down, otherwise it really means that you are not comfortable with others. Even if I have a son, a part of loneliness I will rest, here I leave a big family!

The affection, understanding and bonds of trust that, after a lot of effort and often as a result of numerous failed attempts, were created in the community were perceived as a rare commodity, which could hardly be recreated on leaving the community, when meeting the judgmental gaze of society, less willing to forgive, forget and start over. These fears appeared clearly in the words of an Italian woman, a former drug addict:

In community it made me feel good to be able to speak and be accepted, outside it is not like that. It is very difficult to integrate after a few years on the road, they always look at you the same way and you always have your finger pointed at it, even if you want to rebuild your life.

Faced with fears of being rejected and living in solitude, especially when relationships with the family of origin have been dramatically interrupted, the reaction may be to rebuild relationships with the companions that used to meet on the street, but this decision can be very dangerous. The frequentations of the past, therefore, the pre-existing social networks, can represent a danger of relapse into addiction or the dynamics that in the past led the person to lose their home and job (Bourgois, 2003; Christian and Abrams, 2003; Ravenhill, 2008). As emerged from various interview passages, many guests were aware that to succeed in their exit path, it was important, beyond the economic, to have worked on oneself to strengthen one's character and willpower. A Romanian man, speaking of how he imagined his future, addressed this point:

In the future I see myself as another person, with another mentality, even if the risk of alcohol and stealing are always in front of me; I had such a hard time quitting that now I'm really afraid of starting over. When I get out of here for good I hope to have “something solid”, to find another way, with another company, change everything, work, place where I live, go to live alone and maybe later find a girlfriend, not necessarily an immediately challenging story.

The situation created in the community, of sharing every moment and activity, may initially be suffocating, however, at the end of their journey; users were aware that while in the community they lived a time that was constantly “full” of activities, people, affections and words, what awaits them outside will be many empty times of loneliness and inactivity. While in the community, it was the structure and its organization that somehow provided for their belonging to the context (Putnam, 2006), outside the community they would have to build new bonds themselves and this prospect can be frightening (Cohen and Willis, 1985; Barker, 2012).

A compromise solution, often adopted by the people interviewed, was to invest their time in volunteering, waiting to find a job or new company. The decision to engage in volunteering as a strategy to avoid loneliness also appeared in this Italian woman's words: “Living alone is tiring, also because if you feel alone you look for company outside the home, but it is tiring. Then I'm trying to be a volunteer around the canteen or in a shop”. Volunteering, therefore, represents an opportunity in which it is possible to build bonds of solidarity, even if transitory, with unknown people and this can help in filling that void, of which the others interviewed had spoken.

A different case was that of this Italian man for whom volunteering was more a life choice than a fleeting survival strategy:

I have the dream, but don't laugh! When I go out of here I would like to help the people on the street, because I think you can learn something important from us. One evening they called the coordinator to pick up a lady from the station, who nobody wanted to get her into the car because she smelled really bad. The coordinator asked me to go with her, to help her and the moment we sat in the car I rolled down the windows because that woman smelled really bad, then I realized she was cold and so I pulled them up. Then I didn't hear anything and I realized that you can learn a lot from other people, I would really like to volunteer.

Engaging in volunteering, maintaining collaboration with the community or participating in associative life are activities that are highly encouraged by the community educators, who suggest them to users at the end of their journey. Even if these activities do not provide for the material maintenance of the person, they can be occasions to build bonds and also to continue a path of self-analysis of one's character and experiences (Cohen and Willis, 1985; Barker, 2012).

In any case, the challenge for the future is to build new forms of normality and a rehabilitated self-image that can enhance new bonds. As this Ukraine man said, in relation to his family ties:

My family was used to seeing a joyful husband and father, always ready to react, but later they met another person and did not accept how I had changed. I understood that today I can no longer give 100% but it is not certain that my 50 is not worth as much as that 100, because what little I can give I give it all and I believe that my daughters' affection is left, I think they think about me all the time.

In the path of the new normal, in addition to family ties, which as clearly emerges from this last interview's words represent a fundamental cornerstone, the home also plays a very important role (Munoz et al., 2004; Anderson and Thomson, 2005; Tosi, 2005). These are often rented housing that people have had access to thanks to the support of service operators and after months, if not years, of investment in this direction.

As an Italian man said: “Living means being well, in a place that represents you and that you feel yours. It also means having the opportunity to be involved in the community you live in. Belong”. A stable home, in addition to being the demonstration of a successful social reintegration process, can be the possibility to establish new social networks and become full members of the community.

## Discussion

At the end of phase 1 of the COVID-19 epidemic and in the following months, no deaths were recorded among homeless people and operators, five people were hospitalized without serious symptoms, and every day more than 300 people had a safe place to sleep, spend the day and have a meal.^6^ The enormous commitment shown by the third sector network and by charitable associations was of fundamental importance in protecting a population that, because of their lifestyles and general clinical condition, is to be considered vulnerable (Fitzpatrick, 2013; Barbieri, 2020). At the same time, it filled the absence of the public actor, who in this emergency situation did not seem to take much care to protect the homeless, either in legislating or in organizing services on the ground. If we add to this lack of protection the suspension for an indefinite period of services of fundamental importance, such as access to social housing or entry into the community, the conclusion seems to emerge that public institutions implemented the dynamics of social misrecognition of the homeless (Dubois, 2003; Honneth, 2004; Wacquant, 2009; Muehlebach, 2012). This process of misrecognition was favored by the pandemic approach that characterized the first interventions to combat the crisis, which were focused exclusively on the analysis and treatment of biological factors and were not very attentive to the interconnections between biological vulnerabilities and social needs—a syndemic approach (Horton, 2020a,b). The observations collected in the field in the province of Bergamo are also confirmed in the empirical studies conducted at the national level.^7^

Both the operators and the users were able to react almost immediately to the emergency and to activate forms of social resilience by making use of both the consolidated relational work that had been passed down from previous years and the network of collaboration and shared governance developed between the actors of the third sector and the institutions (Adger, 2000; Keck and Sakdapolrak, 2013). In addition to this, the operators activated an intervention that was as personalized as possible and was aimed at empowering users, sharing decisions and activities, and dedicating a good part of the day to listening and “doing together” (Sennett, 2004). The ability shown by the educational staff to identify, in a short time, an effective method to manage the emergency seems to have arisen from their being accustomed over the years to very flexible and highly stressful jobs. The inheritance of relational work, the network collaboration and the empowering interventions with users were the main resources that helped to make it possible to overcome the crisis. The strategies that were followed made it possible to create the conditions for the feeling of safety that was necessary to maintain the wellbeing of operators and guests. These practices represent an important heritage for the improvement of the social integration of the homeless and the prevention of future health and social crises (Folgheraiter, 2020). However, this great capacity for autonomy demonstrated by the third sector network seems to reaffirm the marginalization of this network and prevent the achievement of a complete process of social recognition of the homeless population.

The analysis of narratives has been confirmed to be a useful tool for a qualitative complement to the social action of people, groups and institutions, and it is also capable of revealing structural characteristics and current transformations (Glaser and Strauss, 1967; Dubois, 2003). The daily experiences lived by operators and users created frames of meaning in which homeless people experienced contrasting sensations every day: on the one hand, they related to the operators and volunteers with whom they had relationships of trust, receiving concrete examples of solidarity, but on the other hand, they were aware of being placed in an economic and institutional context that limited the complete recognition of their protection. Operators also perceived a strong lack of advantage and poor recognition from public institutions; this seemed to be determined by the stigmatization processes affecting the users they assisted. At the same time, they perceived the unique value of their care work.

As the operators interviewed defined themselves as being “accustomed to permanent emergencies”, so the homeless population lives a “normally” insecure life, deprived of the traditional mechanisms of insertion and integration (in respect of the labor market, the welfare state and social ties). In particular, the social housing policies adopted by the Italian state are still unable to respond to the needs of the poor, especially when they are isolated from their family networks (Allen, 2006; Ranci and Pavolini, 2008).

The “normal social insecurity” of the homeless population also seems to have been reaffirmed during the course of the extraordinary COVID-19 syndemic that affected Italy and, in particular, the province of Bergamo, during the spring of 2020. During this period, the homeless population seems to have been forgotten by the institutions and further deprived of their rights, suffering the “official contempt” of public, local and national institutions and forcing the third sector network into an extraordinary, but isolated, reaction (Bifulco and Vitale, 2006; Nolan, 2009).

The social exclusion of homeless people does not seem to be limited to economic deprivation, but also involves a wide range of social, political and cultural processes that feed the disadvantage and exclusion of those affected, progressively eroding their future prospects for life (Anderson and Thomson, 2005; Fitzpatrick, 2013; Petersson, 2017). In this sense, the lack of commitment on the part of the institutions to guaranteed housing seems to legitimize the misrecognition of the homeless population, who personally pay the price for the contradictions of contemporary post-capitalist society (Bauman, 2003; Magatti, 2012). The adoption of neoliberal policies, which still characterizes most western countries (Stiglitz, 2019), means that social insecurity and market mechanisms pervade all spheres of life, including those of protection and fundamental rights, which were seriously endangered during the COVID-19 syndemic (Lusardi and Tomelleri, 2021).

In conclusion, the management of the COVID-19 crisis confirmed the importance of the social context in the development of people's ability to act: that is, the centrality of public responsibility and institutional arrangements to ensure people have tools for participation and free expression (Adger, 2000). The institutional commitment should be realized directly by providing legal instruments and forms of economic intervention in favor of the protection of the most vulnerable. In particular, it is crucial to encourage projects and public initiatives to raise awareness in civil society of the condition of the homeless population. In the absence of a social context capable of offering new opportunities and weaving new social relationships with those who complete the paths of social reintegration, the daily efforts made by the third sector network will continue to be made in vain, and the rates of relapse and chronic state of social malaise will be high.

## Data availability statement

The original contributions presented in the study are included in the article/supplementary material, further inquiries can be directed to the corresponding author.

## Ethics statement

The studies involving human participants were reviewed and approved by Department of Humanities and Social Sciences, University of Bergamo. The patients/participants provided their written informed consent to participate in this study.

## Author contributions

RB contributed to conception and design of the research and wrote all the sections of the manuscript.

## Conflict of interest

The author declares that the research was conducted in the absence of any commercial or financial relationships that could be construed as a potential conflict of interest.

## Publisher's note

All claims expressed in this article are solely those of the authors and do not necessarily represent those of their affiliated organizations, or those of the publisher, the editors and the reviewers. Any product that may be evaluated in this article, or claim that may be made by its manufacturer, is not guaranteed or endorsed by the publisher.

## Footnotes

^1^The Italian Constitution does not provide specific provisions regarding the right to housing; only subsequent jurisprudence links the right of residence directly to human dignity and the protection of the family (Arlotti and Ranci, 2018). In particular, Law no. 1228 of 24.12.1954 provides that registration in the municipal registry is a subjective right (and not a concessionary one) and has to be recognized to all citizens. Foreigners not legally residing in the territory are an exception. Furthermore, the Ministry of the Interior, in the implementation of the Law on public safety no. 94 of 15.07.2009, establishes that once a homeless person has been registered in the resident population registry, the municipalities have to insert their name in the national registry index. This registration allows the homeless person to request the following documents: identity card, health insurance card and residence permit.

^2^ISTAT: Istituto nazionale di statistica. Available online at: https://www.istat.it/it/files//2015/12/Persone_senza_dimora.pdf (accessed April 15, 2022).

^3^The survey was carried out in 2017 by the “PONte” network, which sees the participation of the municipalities of the Bergamo area, charitable associations and the third sector.

^4^The services that intervene in an emergency manner and in response to basic needs are commonly defined as “first reception services”; these include canteens, emergency dormitories and street workers. In the “second reception sector”, on the other hand, we find the therapeutic communities and the social hosing apartments; these resources are intended for people who have embarked on a path to leave street life.

^5^The first European survey on this phenomenon, conducted by Eurofund, dates back to 2002.

^6^ISTAT found that in the province of Bergamo the deaths during the first wave of COVID-19 amounted to 16,368 (62% more than in the previous 5 years), causing a decrease in the natural balance (difference between live births and deaths) of 0.8%. The deaths occurred mainly in nursing homes, hospitals and private homes. Therefore, the absence of deaths in hostels and communities for the homeless represents an excellent result. Available online at: https://www.istat.it/en/archivio/254537 (accessed July 05, 2022).

^7^Available online at: https://www.fiopsd.org/wp-content/uploads/2021/02/Instant_report_2020_short_version.pdf (accessed April 30, 2022).
